# Identification of nuclear genes controlling chlorophyll synthesis in barley by RNA-seq

**DOI:** 10.1186/s12870-016-0926-x

**Published:** 2016-11-16

**Authors:** Nickolay A. Shmakov, Gennadiy V. Vasiliev, Natalya V. Shatskaya, Alexey V. Doroshkov, Elena I. Gordeeva, Dmitry A. Afonnikov, Elena K. Khlestkina

**Affiliations:** 1 Institute of Cytology and Genetics SB RAS, Novosibirsk, Russia; 2 Novosibirsk State University, Novosibirsk, Russia

**Keywords:** Barley, Near-isogenic lines, Chlorophyll synthesis, Albino lemma, Nuclear genes, Gene network, RNA-seq, Differential expression, IonTorrent sequencing platform

## Abstract

**Background:**

Albinism in plants is characterized by lack of chlorophyll and results in photosynthesis impairment, abnormal plant development and premature death. These abnormalities are frequently encountered in interspecific crosses and tissue culture experiments. Analysis of albino mutant phenotypes with full or partial chlorophyll deficiency can shed light on genetic determinants and molecular mechanisms of albinism. Here we report analysis of RNA-seq transcription profiling of barley (*Hordeum vulgare* L.) near-isogenic lines, one of which is a carrier of mutant allele of the *Alm* gene for albino lemma and pericarp phenotype (line i:Bw*Alm*).

**Results:**

1221 genome fragments have statistically significant changes in expression levels between lines i:Bw*Alm* and Bowman, with 148 fragments having increased expression levels in line i:Bw*Alm*, and 1073 genome fragments, including 42 plastid operons, having decreased levels of expression in line i:Bw*Alm*. We detected functional dissimilarity between genes with higher and lower levels of expression in i:Bw*Alm* line. Genes with lower level of expression in the i:Bw*Alm* line are mostly associated with photosynthesis and chlorophyll synthesis, while genes with higher expression level are functionally associated with vesicle transport. Differentially expressed genes are shown to be involved in several metabolic pathways; the largest fraction of such genes was observed for the Calvin-Benson-Bassham cycle. Finally, *de novo* assembly of transcriptome contains several transcripts, not annotated in current *H. vulgare* genome version.

**Conclusions:**

Our results provide the new information about genes which could be involved in formation of albino lemma and pericarp phenotype. They demonstrate the interplay between nuclear and chloroplast genomes in this physiological process.

**Electronic supplementary material:**

The online version of this article (doi:10.1186/s12870-016-0926-x) contains supplementary material, which is available to authorized users.

## Background

Chlorophylls form the most abundant and the most important class of plant pigments, as they play central role in photosynthesis process which is a main source of energy for plants as well as a main source of oxygen in atmosphere of the Earth. In eukaryotic plant and algae cells chlorophyll is located in chloroplasts. Chloroplast is an organoid which, like the mitochondria, contains its own genome sometimes called ‘plastome’ [1]. However, chloroplasts are strongly dependent on the nuclear genome of the plant cell. In angiosperms, plastome contains only approximately 50 protein-coding genes involved in chlorophyll synthesis, photosynthesis and some metabolic processes, as well as tRNA and rRNA genes [2], while the amount nuclear-encoded proteins which are predicted to be targeted to chloroplasts is as much as 2500–3500 (as accessed in *Arabidopsis thaliana*; [3, 4]). In *A. thaliana*, from 1400 to 1500 cyanobacterial proteins have been identified, of which about a half are targeted to chloroplasts [5]. It was suggested that about 4500 *A. thaliana* genes descend from cyanobacterial genomes, however, only about 1300 of them are predicted to have their products targeted to chloroplasts [6].

Transcription of chloroplast genome is provided by two types of RNA polymerase – a nuclear-encoded plastid RNA-polymerase (NEP) and plastid encoded plastid RNA-polymerase (PEP). PEP is an RNA polymerase of bacterial design, and it uses sigma-factors for transcription initiation, which are nuclear-encoded [7]. Chloroplast genes can be divided into three groups – class I genes have promoters that are only recognized by PEP, class II have promoters recognized by both enzymes, and class III are only transcribed by NEP [8]. Most genes in plastome have promoters that can be recognized by both types of polymerase [9].

Chloroplast genes control is mostly post-transcriptional. It is regulated by nuclear-encoded proteins, and in some cases a single nuclear-encoded protein is required specifically for controlling of expression of a single chloroplast-encoded gene [10].

All of the above suggests that a sophisticated coordination of plastome and nuclear genome is required for proper development and functioning of chloroplasts. Such coordination is formed by anterograde signaling – from nucleus to chloroplast – and retrograde signaling – from chloroplast to nucleus [11]. Intermediates of chlorophyll synthesis pathway, namely protoporphyrin IX, serve as signaling molecules and are transported from chloroplast to nucleus [12]. On the other hand, a number of authors disagree with the role of protoporphyrin IX as a signaling molecule [13, 14]. This leads to a conclusion that mechanisms of nucleus-to-chloroplast and chloroplast-to-nucleus signaling are poorly understood.

Although a number of mutant phenotypes with full or partial chlorophyll deficiency have been described, genes responsible for such phenotype formation are largely unknown. Barley (*H. vulgare* L.) *Alm* gene (chromosome 3H; [15]) determining albino lemma and pericarp has not been sequenced yet, and the target or regulatory genes for *Alm* remain unknown. Near-isogenic lines (NILs) are the proper models for isolation of genes underlying phenotypic variation by transcriptomics, proteomics or metabolomics approaches (reviewed in [16]). Availability of barley genome reference sequence [17] facilitates application of WGS-based approaches (such as RNA-seq) for investigating genetic networks responsible for phenotypic variation. RNA-seq approach has been already employed successfully to uncover the history of wild barley domestication and distribution [18], for studying the gene family of barley aquaporines [19] or creating SNP maps of barley [20].

In the current study, RNA-seq was exploited for identification of genes involved in tissue-specific albinism using near-isogenic lines differing by the *Alm* gene allelic state.

## Results

### Phenotypic characterization of the near-isogenic lines and pleotropic effect of Alm

**Fig. 1:**
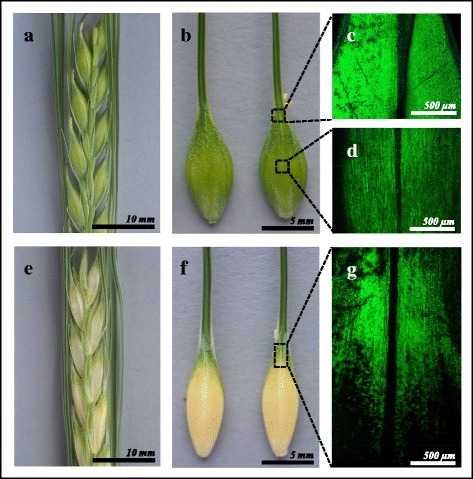
The spike phenotype of Bowman (**a**-**d**) and i:Bw*Alm*(**e**-**g**). **a**, **b**, **e**, **f** appearance at visible light. **c**, **d**, **g** chlorophyll fluorescence patterns of selected areas

The i:Bw*Alm* near-isogenic line is characterized by albino lemma (base and the central part), however the upper part of the lemma and awns are green. Bowman lemma and awn have a green color (Fig. 1). Chlorophyll fluorescence is observed in the central part of the lemma in Bowman, while it is absent in the central part of i:Bw*Alm* lemma(Fig. 1d). Chlorophyll fluorescence is also observed in lemma-awn transition point of Bowman (Fig. 1c). In i:Bw*Alm*,the analysis of the fluorescence pattern in transition point between the white and green zones (Fig. 1f) detected an alternation of cells containing a population of fluorescing chloroplasts and lacking them (Fig. 1g). While moving in the basal direction the number of detected fluorescent cells decreases to extinction (Fig. 1g). The appearance and fluorescent pattern in awns (and other similarly colored parts of plant such as mid-stem and leaf blade) did not reveal differences between Bowman and i:Bw*Alm*.

**Fig. 2:**
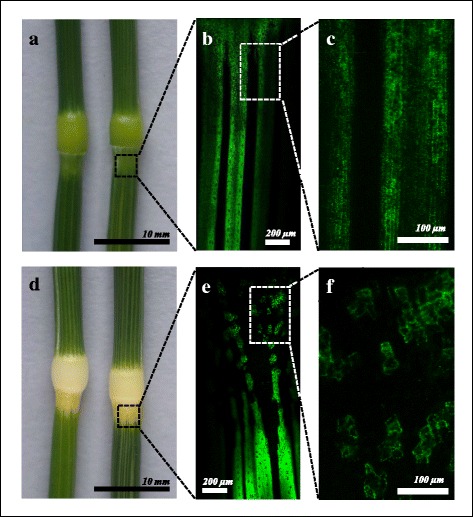
The stem node phenotype of Bowman (**a**-**c**) and i:Bw*Alm* (**d**-**f**). **a**, **d** appearance at visible light. **b**, **c**, **e**, **f** chlorophyll fluorescence pattern of selected areas at low and high magnifications

Pleutropic effect of *Alm* was observed on auricles (and adjacent leaf blade regions), stem node (and adjacent stem regions; Fig. 2) and sheath of the first leaf of the i:Bw*Alm* line. In Bowman, all these parts of plant are green. Along the surface of stem both lines have the green strands with bright chlorophyll fluorescence (Fig. 2), however in the i:Bw*Alm* node adjacent region these strands flat out, and a fragmentation of fluorescent strands into the loosely spaced islands is detected (Fig. 2e, f). The shape of patterns of cell population containing fluorescing chloroplasts in this zone differs between Bowman and i:Bw*Alm*. In the Bowman plants cells arranged in files (Fig. 2c). In i:Bw*Alm* cells with fluorescence form irregular patterns, no cell files are observed (Fig. 2f).

Thus, the *Alm* gene results in vanishing of a chloroplasts population with chlorophyll in lemma, pericarp, auricles with adjacent leaf blade region, stem node with adjacent stem region and sheath of the first leaf. The alternation of the patterns of cells containing a population of fluorescing chloroplasts and cells without fluorescence signal is evident in transition region between the white and green zones (Figs. 1g and 2f). Difference between i:Bw*Alm* and Bowman in the cell arrangement (Fig. 2c, f) demonstrates that *Alm* gene, in addition to effects on chloroplasts, results in modification of patterning of cells with active chloroplasts in pericarp and auricles.

### qPCR gene differential expression validation

**Fig. 3:**
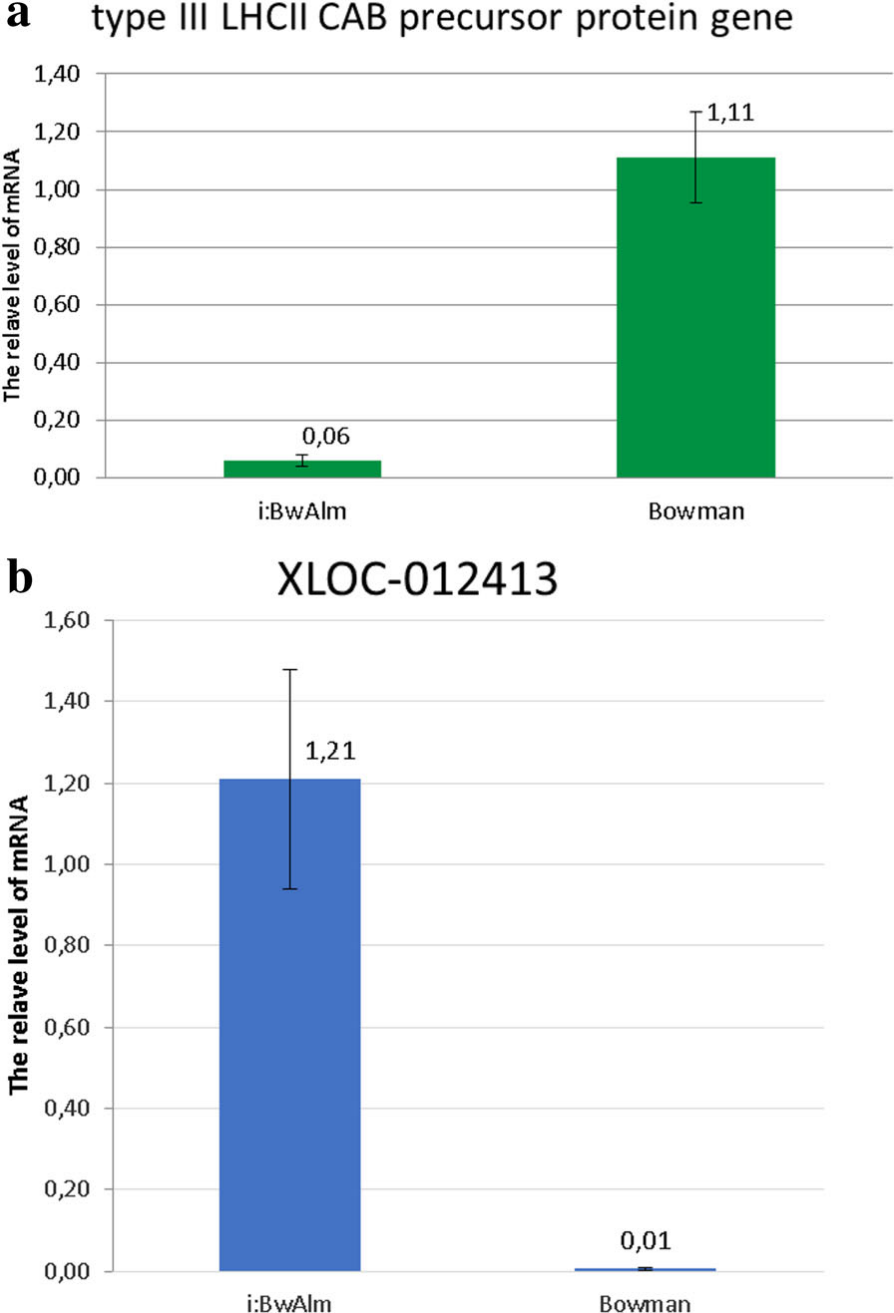
Changes in transcription level of genes MLOC_17002 (**a**) and XLOC_012413 (**b**) as obtained with qPCR experiment

The qRT-PCR-based evaluation of transcription of two genes in the spikelets of the NILs is summarized in Fig. 3. The first is gene MLOC_17002 that encodes Type III Light-harvesting complex II Chlorophyll a/b-binding precursor protein. Its expression level estimated from RNA-seq data decreased in i:Bw*Alm* plants more than 45-fold (corrected with Benjamini-Hochberg procedure *p* =0.002). The second is a XLOC_012413 transcript that, according to Gene Ontology analysis, is functionally connected to vesicle transport. Its expression level estimated from RNA-seq data increased in i:Bw*Alm* plants more than 1000-fold (corrected *p* = 0.002). According to RT-PCR data Type III LHCII CAB precursor protein gene transcript was abundant in Bowman and significantly decreased (18.5 fold) in albino plants (Fig. 3a). XLOC_012413 transcript was poorly detectable in Bowman, however its expression level increased significantly (121 ford) in spikelets of i:Bw*Alm* plants (Fig. 3b). The difference between expression levels estimated by RT-PCR for these two genes is *p* = 0.033 for MLOC_17002 gene and *p* = 0.008 for XLOC_012413 gene according to *t*-test.

### Libraries quality assessment

28481151 short reads in six pooled libraries were produced as raw sequenced data. Each library was examined for quality control, adapter sequences were trimmed and reads with insufficient length or too low mean quality were removed, which resulted in discarding of 7.4 % to 13.4 % of reads in five of six libraries, and removal of 40 % sequences in library Bowman (2) (Table 1).

**Table 1:** Read statistics for i:Bw*Alm* and Bowman libraries

	i:Bw*Alm*(1)	i:Bw*Alm*(2)	i:Bw*Alm*(3)	Bowman(1)	Bowman(2)	Bowman(3)
Library size (reads)	4596395	3056413	5794644	4122599	4023501	6887599
Adapters trimmed	336370 (7,3 %)	197932 (6,3 %)	434647 (7,5 %)	273892 (6,6 %)	189062 (7,4 %)	518737 (7,5 %)
Reads filtered by quality	542101 (11,79 %)	409621 (13,4 %)	429283 (7,41 %)	330121 (8 %)	1628646 (40,5 %)	236444 (3,43 %)
Library size after pre-processing	4054279	2646786	5365347	3792465	2394836	6651154
Mean read length after filtering	171	208	181	177	128	202

**Fig. 4:**
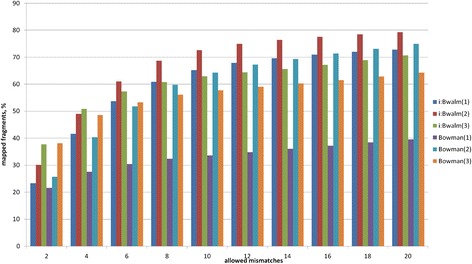
Dependence of the number of mapped reads with respect to mismatch parameter for TopHat2 alignment of short read libraries. The x-axis indicates the number of allowed mismatches for the mapping; the y-axis shows the percent of mapped reads for each library

Next, we performed analysis of the mapping reads from the six libraries to estimate the optimal value of the mismatch parameter. Mapping reads to the reference genome of *H. vulgare* was performed with TopHat2 tool ten times, with increasing number of allowed mismatches from 2 to 20 as described in the Methods section. The resulting FPKM counts for each transcribed genome fragment are listed in Additional file 1. We observed an increase in percentage of fragments mapped to genome in each library with increasing mismatch number (Fig. 4). In five of six libraries proportion of mapped reads ranged from 23 % to 38 % (2 mismatches allowed) to 64 % to 79 % (20 mismatches allowed); for the Bowman (1) library percentage changes from 21 % to 39 % for alignment with 2 and 20 mismatches, respectively (Fig. 4).

**Fig. 5:**
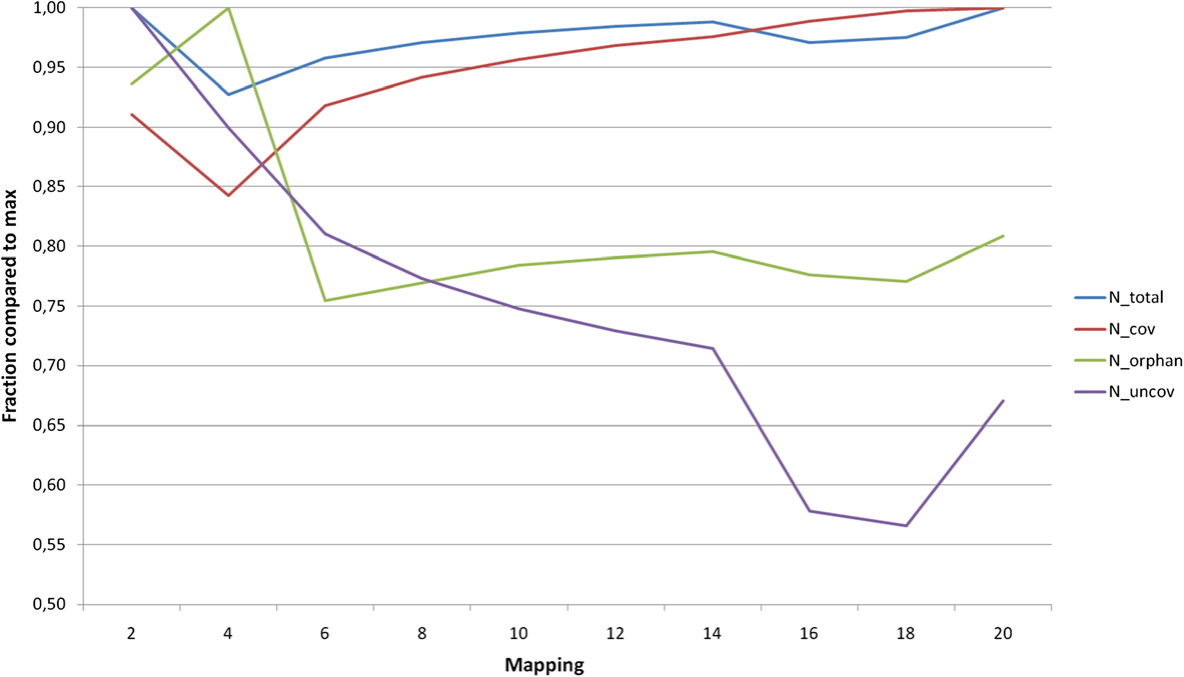
Dependence of the number of mapped reads to the different types of expressed genome fragments on allowed mismatches. See Methods for more information

Additionally we monitored changes of four characteristics of genome coverage quality for six libraries (see the Methods section) with respect to mismatch number. The ratio of these values to their maxima in ten TopHat2 alignments is shown in Fig. 5. This plot (Fig. 5) demonstrated that number of mapped reads increases with increase in number of allowed mismatches. However, at mismatch values greater than 12 the number of matched reads saturates its maximal value. Similarly, N_uncov_ drops with increase in number of allowed mismatches, and N_orphans_ is reduced in alignments with six or more allowed mismatches compared to alignments with two and four allowed mismatches. Combination of these three parameters is believed to be optimal for alignment with 18 allowed mismatches.

Aligning reads to the reference genome with STAR program resulted in successfully mapping of 72 to 83 % of reads for five libraries out of six, with Bowman (1) library having only 46 % of mapped reads (Table 2).

**Table 2:** Metrics of libraries mapping to *H. vulgare* reference genome using STAR tool

Library	Uniquely mapped reads %	Average mapped length	Multi-mapped reads %
I:Bw*Alm*(1)	82,5	158	3,1
I:Bw*Alm*(2)	83,3	196	3,1
I:Bw*Alm*(3)	77,2	169	2,2
Bowman(1)	46	154	3,1
Bowman(2)	72,4	113	2,9
Bowman(3)	74,4	189	1,7

After examination of considered parameters, the mapping with 18 allowed mismatches was taken into further analysis. A total of 14.9 million of reads were mapped to the reference genome from all six libraries. Cufflinks pipeline identified 33537 genome fragments that are covered by short reads from at least one library and/or are annotated in the current genome assembly.

**Fig. 6:**
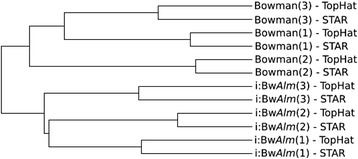
Libraries clustering. Six mappings performed with TopHat2 and six mappings performed using STAR were clusterized. Clustering was performed with UPGMA method basing on the count numbers of each transcribed genome fragment

Next, we compared the coverage of the *H. vulgare* transcripts by reads from each of the libraries. We generated tables of counts indicating the transcript coverage for 24158 genomic regions from *H. vulgare* genome annotation by cuffnorm tool, 18820 of which passed out filtering criteria. Results of clustering of six libraries with respect to the similarity of coverage of these regions are shown in Fig. 6. The tree diagram demonstrates that estimates of coverage for *H. vulgare* genes form two large clusters corresponding to two contrast genotypes irrespectively to the alignment methods and sample replicate. Detailed analysis of the diagram demonstrates that the read counts estimates performed by TopHat2and STAR tools for the same replicate are closer than those for different replicates are.

### Analysis of transcript abundance

**Fig. 7:**
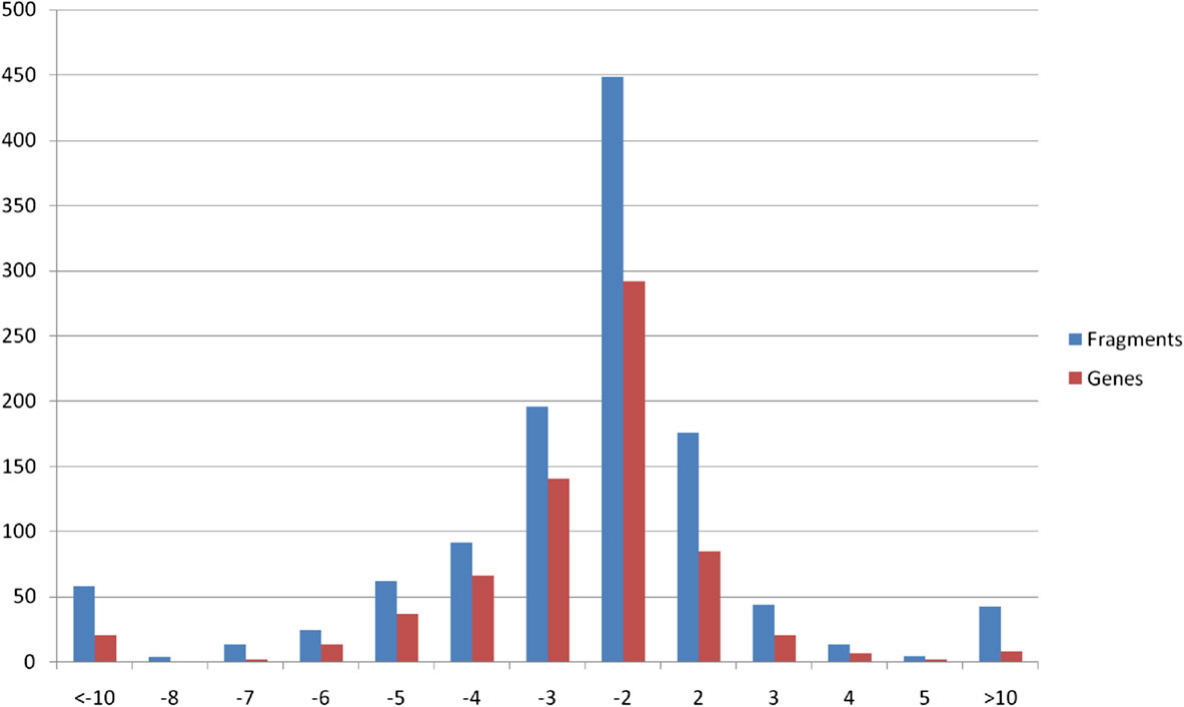
Distribution of genome fragments and genes with difference in expression levels. Horizontal axis shows a log_2_FC of gene expression in line i:Bw*Alm* compared to line Bowman. Vertical axis shows a number of genes (*red*) or genome fragments (*blue*) with respective changes in expression level

One thousand seven hundred and eighty-six genome fragment identified by cufflinks pipeline (5 % of total number of transcribed genome fragments) showed changes in expression levels with |log_2_FC| > 2. List of transcribed fragments and genes with expression level changes greater than four-fold is presented in Additional file 2. One thousand two hundred and eighty-four of those fragments have a lower level of expression in i:Bw*Alm*, while 502 fragments have a higher expression level in i:Bw*Alm*in comparison with Bowman. Among those fragments are 1140 genes annotated in current barley genome assembly. Eight hundred and four of those genes have a decrease in expression level in i:Bw*Alm*, 336 genes have an increase in level of expression in i:Bw*Alm*. List of genes with changes in expression level contains 149 genes with known products, of which 132 have a lowered expression rate in i:Bw*Alm* and 17 have a higher expression rate in i:Bw*Alm* line in comparison with Bowman. Distribution of genome fragments and genes with difference in expression levels is shown at Fig. 7. Genes with known functions and higher expression levels in i:Bw*Alm* are listed in Table 3 and described below in details.

**Table 3:** Genes with known functions with higher expression levels in i:BwAlm line

Gene	Product	Log_2_FC	*p*-value
MLOC_33278	DNA helicase homolog, putative	>10	0.32
MLOC_74627	Cysteine proteinase	5.45	0.48
MLOC_65942	iron-regulated solute carrier family protein	4.82	1
MLOC_66447	Pectinesterase	4.77	0.52
MLOC_5895	Lipase/lipoxygenase PLAT/LH2 family protein	4.75	0.6
MLOC_22576	plant invertase/pectin methylesterase inhibitor superfamily protein	4.22	0.51

Values of expression fold change in form of base 2 logarithm and corrected with Benjamini-Hochberg procedure are provided for each gene

Genes MLOC_33278 and MLOC_74627 encode a putative homolog of DNA helicase and cystein proteinase, respectively.

Gene MLOC_65942 encodes iron-regulated solute carrier (SLC) family protein. The majority of the SLC transporters are secondary active transporters, such as exchangers, symporters, and antiporters, for which transport is driven by various energy coupling mechanisms [21].

Gene MLOC_66447 encodes pectinesterase protein. This enzyme catalyzes demethylation of pectin. Pectin is a component of cell wall of land plants, and in plant cell pectinesterase is an ubiquitous enzyme associated with cell wall [22]. It is known to participate in a number of processes, including pollen tube growth and pollen tetrad separation [23]. Gene MLOC_22576 encodes a protein of pectin mythylesterase inhibitor superfamily.

Gene MLOC_5895 encodes a protein of PLAT/LH2 family. It is a family of proteins that contain PLAT (Polycistin-1, Lipoxygenase, Alpha-Toxin) or LH2 (Lipoxygenase homology) domain [24]. This domain binds Ca^2+^ ions [25].

The most abundantly transcribed genes in Bowman are chloroplast genes encoding rubisco small chain and ribosomal S12 protein and nuclear genes that encodes rubisco small chain (Table 4). These genes have a lower level of expression in i:Bw*Alm*. Other genes with high level of expression in Bowman include sucrose synthase, tubulin α-3 chain, S-adenosylmethinonine decarobxulase proenzyme, phenylalanine ammonia-lyase, ATP synthase subunit β, translationally-controlled tumor protein homologue and two uncharacterized proteins. These genes also have a very high levels of expression in i:Bw*Alm* line (Table 4).

**Table 4:** Genes with highest levels of expression in Bowman and i:Bw*Alm*

Gene	Product	I:Bw*Alm*	Bowman
EPlHVUG00000010074	Rubisco large chain	11,8	185,3
MLOC_64679	Rubisco small chain cloroplast	6,5	95,6
EPlHVUG00000010038	Ribosomal S12	30,3	60
MLOC_50162	Sucrose synthase	81,3	56,4
MLOC_69930	Tubulin alpha-3 chain	74,3	48,8
MLOC_12446	Uncharacterized gene	94,3	46,1
MLOC_69140	S-adenosylmethionine decarboxylase proenzyme	86,7	44,4
MLOC_64900	Phenylalanine ammonia-lyase	49,5	35,6
MLOC_77840	Translationally-controlled tumor protein homologue	43,8	32
MLOC_76000	Uncharacterized gene	36	31,8
MLOC_7079	ATP synthase subunit beta	54	31

Table shows numbers in thousands of library fragments mapped to the respective genes in i:Bw*Alm* and Bowman

### Differential expression identification

**Fig. 8:**
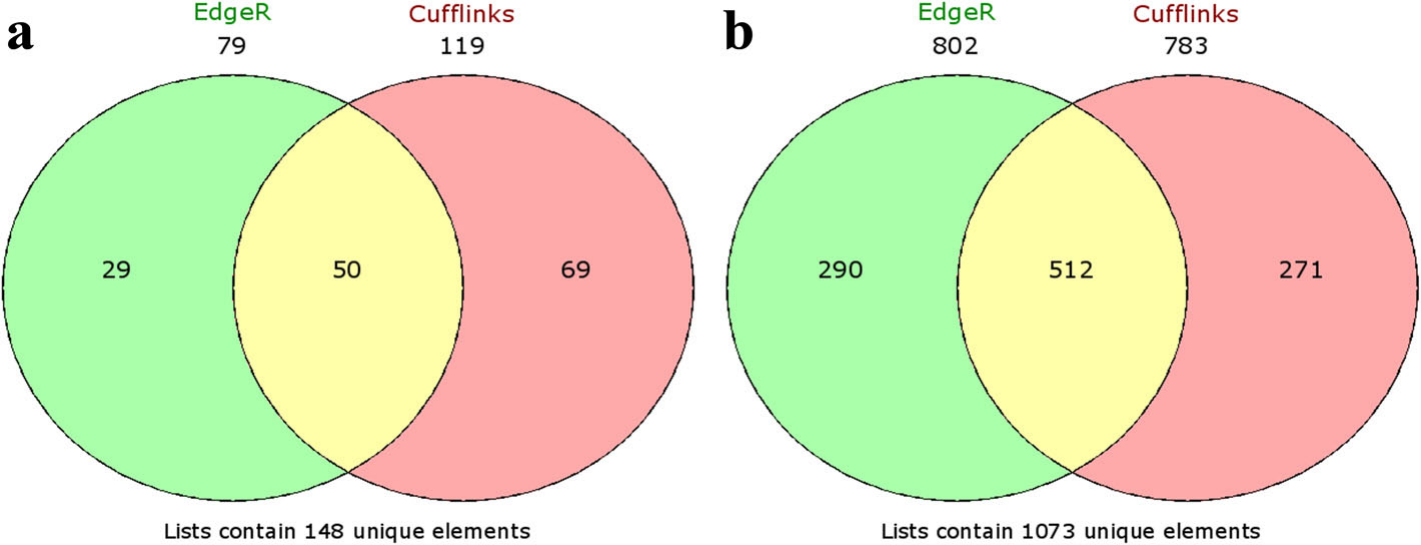
Consistency between edgeR and Cufflinks identification of differentially expressed genes. Consistency of list of genes with lower level of expression in line i:Bw*Alm* is shown on the diagram **a**. The diagram **b** shows consistency of lists of genes with higher level of expression in line i:Bw*Alm*. Genes detected with edgeR are shown in green, genes detected with Cufflinks are shown in *red*

We used two tools to identify differentially expressed genes between i:Bw*Alm* and Bowman lines. Using Cufflinks pipeline we detected 902 genome fragments with differential expression (see methods), of which 119 had a higher level of expression in i:Bw*Alm* plants, and 783 had a higher level of expression in Bowman line plants. Implementing EdgeR package for R, we detected 79 and 802 fragments with higher and lower expression levels in i:Bw*Alm* plants, respectively (Fig. 8). 512 fragments with increased expression and 50 fragments with decreased expression in line i:Bw*Alm* were detected with both tools.

**Fig. 9:**
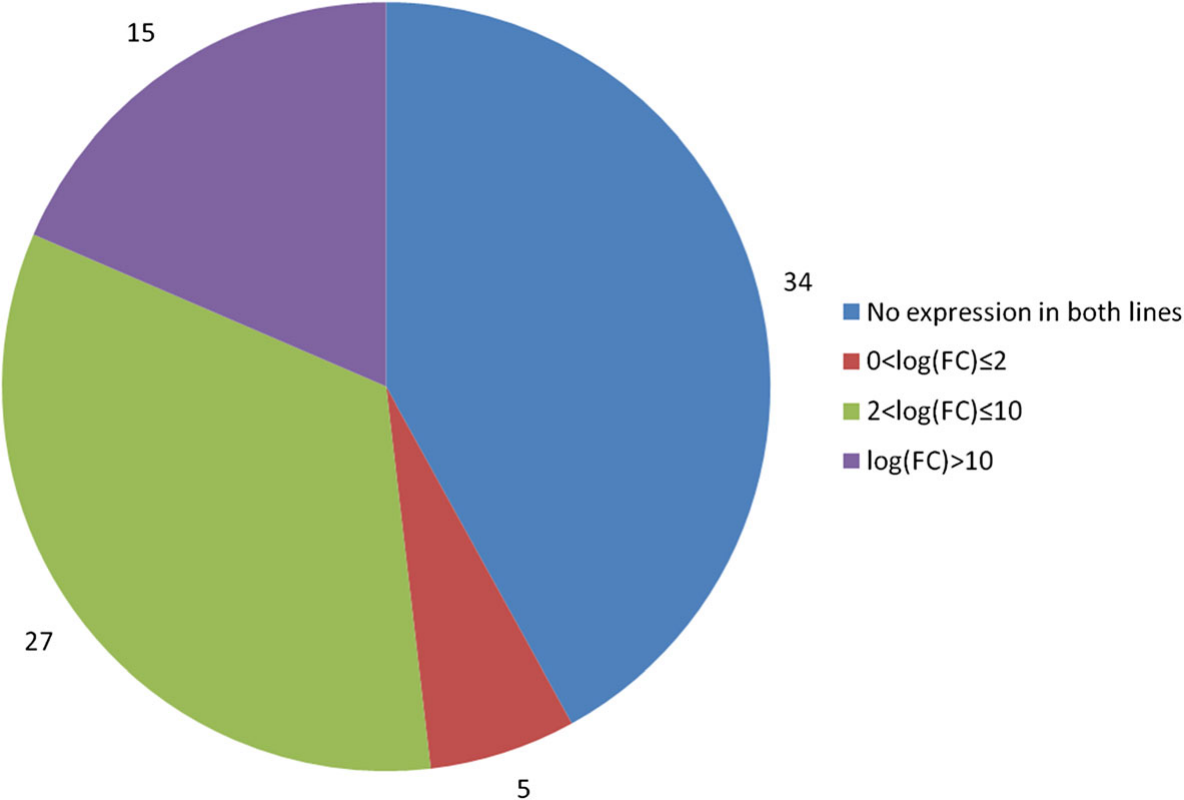
Distribution of coverage level changes among plastid operons. Operons were categorized into four groups according to their expression levels in two barley lines. The group named log(FC) > 10 corresponds to genes that have zero expression in i:Bw*Alm* line and non-zero expression in line Bowman

Thus, 1221 genome fragments are revealed to have differential expression (|log_2_FC| > 1, *p* < 0.05 after correction with Benjamini-Hochberg procedure), which comprises approximately 3.6 % of all transcribed fragments. Among those there are 1073 genome fragments with lower level of expression in i:Bw*Alm*. 694 of those fragments are genes annotated in barley genome current assembly, 115 have a known protein product. 148 genome fragments have differential expression with higher level of expression in i:Bw*Alm*. 67 of those fragments are genes annotated in *H. vulgare* genome assembly. 4 of those genes have known protein products – gene MLOC_61063 that encodes 3-ketoacyl-CoA synthase, gene MLOC_63089 that encodes asparagine synthetase, gene MLOC_51356 encoding leucine-rich receptor-like protein kinase family protein, and gene MLOC_3822 encoding non-specific lipid transfer protein. Figure 9 shows discrepancy between a set of genes with higher and lower levels of expression in line i:Bw*Alm.*

Furthermore, cufflinks pipeline identified 571 genome fragments as having differential isoform expression (see Methods) with *p*-value corrected through Benjamini-Hochberg procedure less than 0.05. Of these fragments, 282 are genes annotated in current assembly of barley genome. 83 of these genes have known functions. List of genes with differentially expressed isoforms includes gene MLOC_50938 encoding tetratricopeptide-like superfamily protein, gene MLOC_60122 encoding thylakoid rhodanese-like protein, gene MLOC_3822 encoding non-specific lipid transfer protein and genes MLOC_945 and MLOC_20487 that encode chlorophyll a-b binding proteins. This list also includes 16 fragments localized on plastid genome. Genome fragments with differential isoform expression are listed in supplementary Table 2.

### Chloroplast gene expression analysis

Dissimilarity between coverage of chloroplast genome fragments was observed in results of cufflinks library reads mappings. According to the results obtained, 34 genome fragments have zero level of expression in both lines. Among those fragments are 23 genes of tRNA, genes that encode two ribosomal proteins, photosystem II protein Z, two RNA-polymerase subunits, four cytochrome proteins, NADH-plastoquinon oxidoreductase subunit 6 and gene *ycf15* that encodes hypothetical chloroplast protein. 15 genome fragments that are expressed in Bowman line have zero level of expression in i:Bw*Alm* line. Among these fragments are genes that encode three photosystem I proteins, two photosystem II proteins, ATP synthase CF0 subunit IV, four NADH-plastoquinon oxidoreductase subunits, chloroplast envelope membrane protein, photosystem I assembly protein Ycf4, gene of 5S ribosomal RNA and three genes of tRNA. Additional file 3 lists expressed chloroplast genome fragments, respective FPKM values for i:Bw*Alm* libraries and Bowman libraries and log_2_ values of expression fold change.

**Fig. 10:**
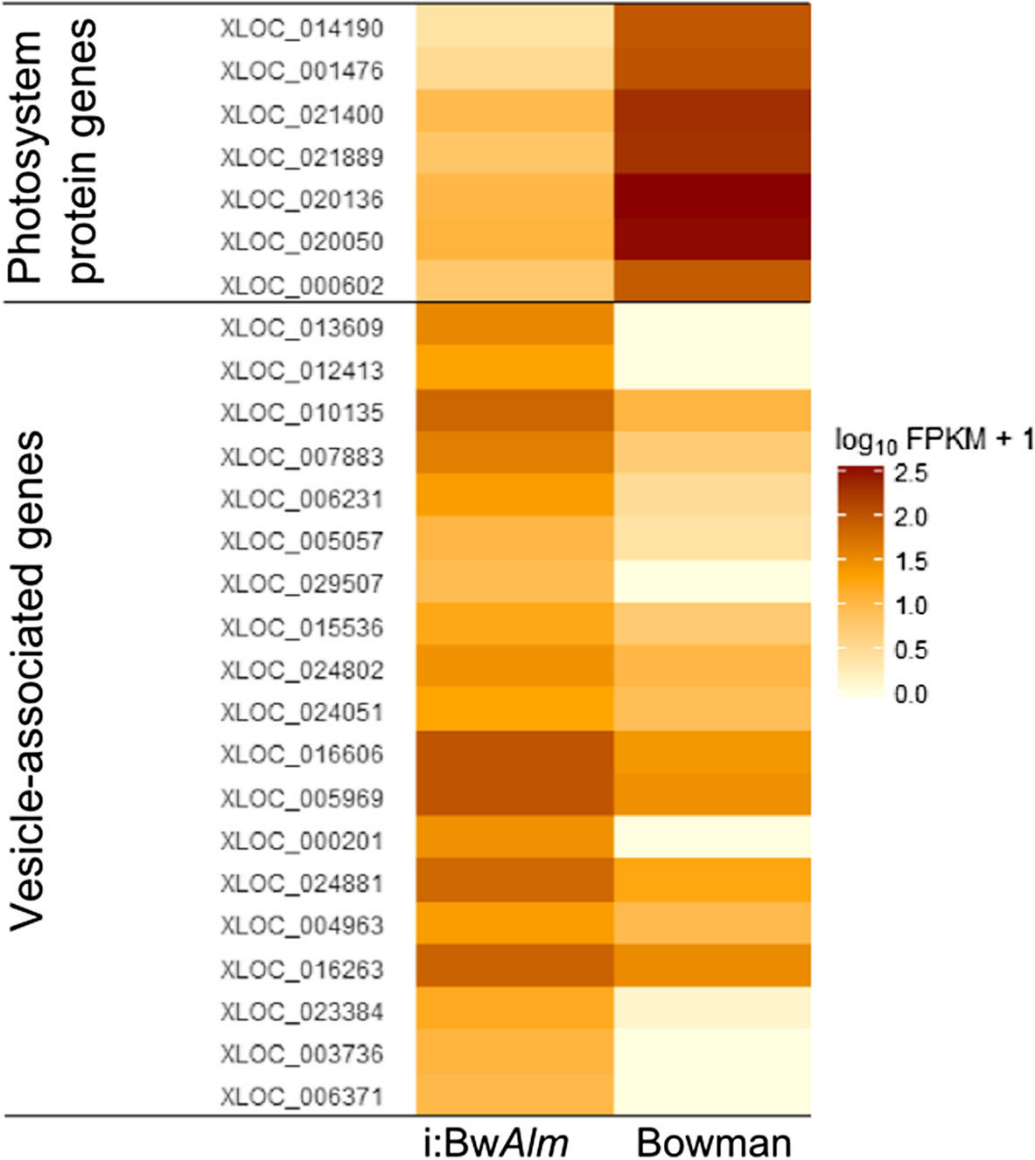
Changes of expression levels of selected gene sets between two barley lines. Genes that encode some of the photosystems I and II proteins and genes that have a functional association with vesicle transport according to the results of GO enrichment analysis were taken as an example. Genes encoding photosystem proteins have a lower level of expression in i:Bw*Alm* line. Genes associated with vesicle transport have a higher level of expression in i:Bw*Alm* line

All chloroplast genome fragments that have nonzero coverage in i:Bw*Alm* have higher coverage levels in Bowman line. Figure 10 illustrates a number of operons with one of four levels of expression change: ‘Zero transcription’ for operons with no transcription observed in both studied lines, ‘log(FC) < 2’ for operons with less than four-folds change of transcription level, ‘log(FC) > 2’ for operons with change in transcription level from four-folds to 368-fold, and ‘log(FC) > 10’ for operons with zero transcription in line i:Bw*Alm* and sufficiently high level of transcription in line Bowman.

A plastid genome fragment that contains 17 genes has the highest among all plastid fragments level of coverage in i:Bw*Alm* line. Of those 17 genes, 13 are genes of ribosomal proteins. Four other genes encode photosystem II protein N, translational initiation factor 1, RNA-polymerase alpha-subunit and NADH-plastoquinon oxidoreductase subunit 2. List of other fragments that have high coverage in i:Bw*Alm* line includes four ribosomal RNA coding genes, three photosystem II protein genes, rubisco large chain gene, ATP synthase CF0 subunits I and III genes, ATP synthase CF1 alpha subunit gene and ribosomal protein S12 gene.

### Gene ontology analysis of differentially expressed genes

We analyzed the distribution of the putative protein products GO-terms annotation in three categories: biological function, molecular functions and cellular compartment localization. The most significantly presented gene ontology terms related to molecular function, biological processes and cellular compartment localizations attributed to putative protein products of genes with higher level of expression in Bowman are shown in Table 5.

**Table 5:** Most represented GO terms associated with differentially expressed genes

Category	GO term	Number of genes associated	*p*-value
Biological process	Photosynthesis	11	6.8e-05
	porphyrin biosynthetic process	6	9.5e-05
	cofactor metabolic process	9	0.00011
Molecular function	structural constituent of ribosome	28	0.00033
	structural molecule activity	34	0.0002
	serine-type endopeptidase activity	5	0.076
Cell localization	Plastid	223	2.2e-50
	intracellular membrane-bounded organelle	303	2e-13
	membrane-bounded organelle	304	1.2e-13

For genes with higher expression level in i:Bw*Alm* only GO terms associated with cell localization were identified. Nineteen out of 65 genes were associated with following terms: ‘vesicle’, ‘membrane-bound vesicle’, ‘cytoplasmic vesicle’, ‘cytoplasmic membrane-bound vesicle’ at corrected *p* = 0.00128 for each of the terms.

Additional files 4 and 5 show GO terms associated with biological processes and cellular components for genes with lower level of expression in i:Bw*Alm* line, respectively. Additional file 6 illustrates GO terms associated with cellular components for genes with higher expression level in line i:Bw*Alm*.

### Pathway analysis for differentially expressed genes

Six hundred and ninety-four genes with changes of expression level were analyzed in respect to their involvement in metabolic pathways. ‘Omics data mapping’ tool of PlantCyc database recognized 132 of these genes. Seventeen metabolic pathways included at least two genes with significant expression level change. Five pathways with highest numbers of genes with different expression levels are listed in Table 6. Other pathways are: sucrose biosynthesis I (from photosynthesis), triacylglycerol degradation, NAD/NADH phosphorylation and dephosphorylation, homogalacturonan degradation, aerobic respiration III (alternative oxidase pathway), gluconeogenesis I, glycolysis I (from glucose 6-phosphate), CDP-diacylglycerol biosynthesis I, triacylglycerol biosynthesis, phosphate acquisition, chlorophyllide a biosynthesis I (aerobic, light-dependent).

**Table 6:** Barley metabolic pathways that involve genes with differential expression between Bowman and i:Bw*Alm* lines

Pathway	Gene IDs	Protein product names	Log_2_(FC)	*p*-value
Calvin-Benson-Bassham cycle	MLOC_58999	Phosphoribulokinase	−3,89	0.0001
	MLOC_44795	Ribulose bisphosphate carboxylase small chain	−4,67	0.002
	MLOC_34272	Uncharacterized protein	−2,50	5e-05
	MLOC_61558	Ribulose bisphosphate carboxylase large chain	−3,35	0.014
	MLOC_64679	Ribulose bisphosphate carboxylase small chain	−3,86	0.007
	MLOC_76055	Phosphoglycerate kinase	−2,08	0.002
	MLOC_44511	Predicted protein	−4,35	0.002
	MLOC_62317	high cyclic electron flow 1	−3,31	0.002
	MLOC_19670	Uncharacterized protein	−2,17	0.002
	MLOC_21811	Ribulose bisphosphate carboxylase small chain	−3,72	0.0005
Photosynthesis light reactions	MLOC_77860	Predicted protein	−1,87	0.02
	MLOC_24730	Uncharacterized protein	−4,95	0.002
	MLOC_64318	Predicted protein	−2,42	0.006
	MLOC_69460	photosystem I subunit O	−5,97	0.002
	MLOC_70480	Uncharacterized protein	−4,29	0.01
	MLOC_70835	Cytochrome b6-f complex iron-sulfur subunit	−2,64	0.002
	MLOC_71570	Uncharacterized protein	−4,74	0.002
Rubisco shunt	MLOC_34272	Uncharacterized protein	−3.51	0.002
	MLOC_44795	Ribulose bisphosphate carboxylase small chain	−5.04	0.002
	MLOC_58999	Phosphoribulokinase	−4.02	0.0001
	MLOC_61558	Ribulose bisphosphate carboxylase large chain	−4.47	0.014
	MLOC_64679	Ribulose bisphosphate carboxylase small chain	−4.27	0.007
	MLOC_21811	Ribulose bisphosphate carboxylase small chain	−3,72	0.0005
Adenosine ribonucleotides *de novo* biosynthesis	MLOC_15467	Adenylosuccinate synthetase	−2,82	0.002
	MLOC_24862	Uncharacterized protein	−2,39	
	MLOC_40997	Uncharacterized protein	−2,53	0.02
	MLOC_58581	ATPase F1 complex gamma subunit protein	−2,29	0.002
	MLOC_74679	ATP synthase delta-subunit gene	−3,14	0.002
	MLOC_75851	Uncharacterized protein	−1.57	0.007
Chlorophyllide a biosynthesis I (aerobic, light-dependent)	MLOC_44116	Predicted protein	−2.94	0.02
	MLOC_81101	Predicted protein	−2.17	0.002
	MLOC_11877	Uncharacterized	−2.55	0.002
	MLOC_80913	Uncharacterized	−3.41	0.002
	MLOC_5142	Predicted protein	−1.57	0.002
	MLOC_49468	Magnesium-protoporphyrin IX monomethyl ester	−4.14	0.002

Values of logarithm of fold change and *p*-values (corrected with Benjamini-Hochberg procedure) are provided for each gene

**Fig. 11:**
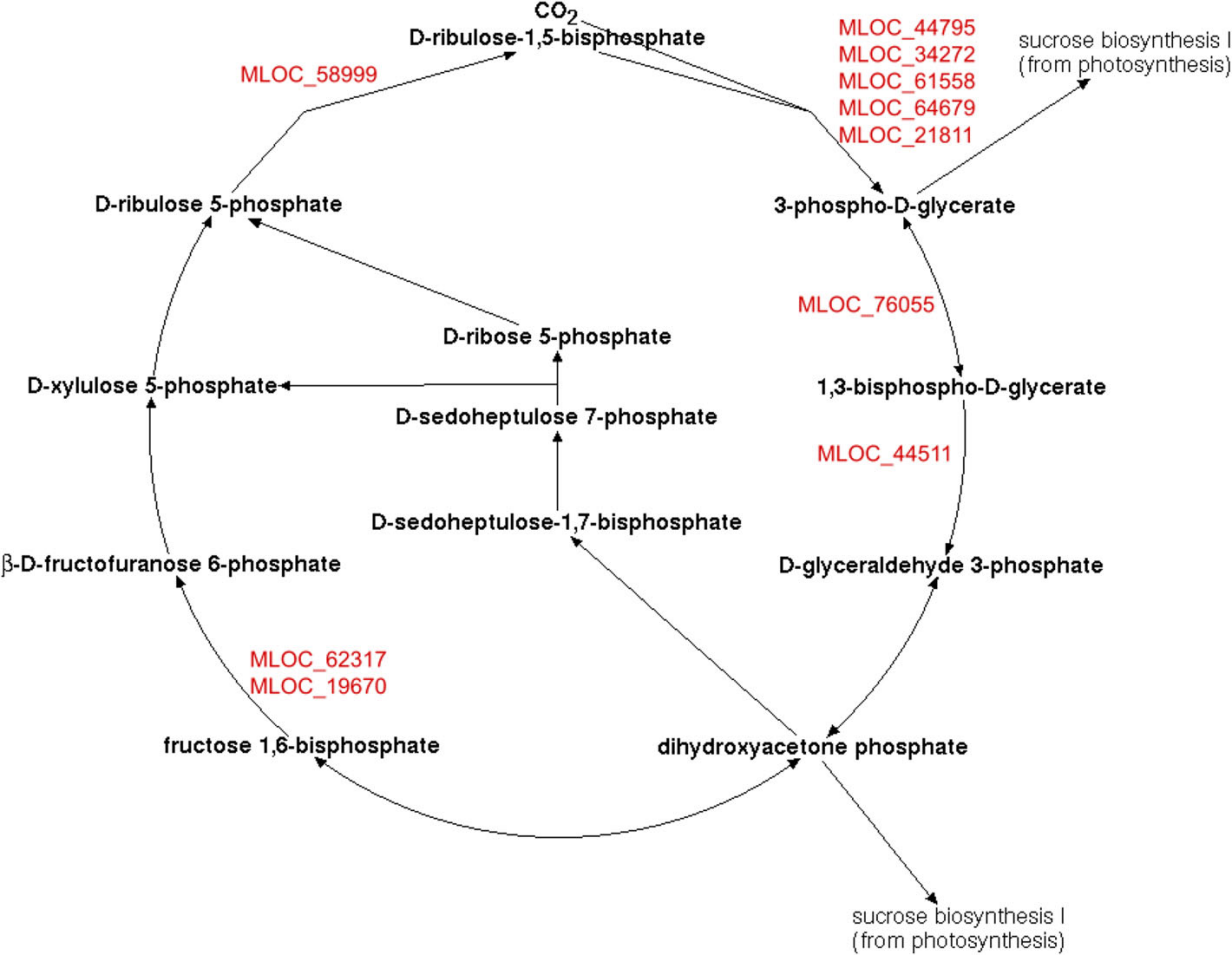
Participation of differentially expressed genes in Calvin-Benson-Bassham cycle pathway. The scheme of pathway is taken from PMN database. Genes with higher level of expression in Bowman line are marked in *red*

The ‘Calvin-Benson-Bassham circle’ pathway that includes a highest number of differentially expressed genes is shown in the Fig. 11. It is related to ‘sucrose biosynthesis I (from photosynthesis)’ pathway that has four genes with different levels of expression. For gene MLOC_65956, which encodes an uncharacterized protein, differential expression was not confirmed. The other three genes included in a pathway are MLOC_62317, MLOC_19670 and MLOC_76055, which are also included in Calvin-Benson-Bassham cycle, and have a confirmed differential expression.

The step of Calvin-Benson-Bassham cycle that has the highest number of differentially expressed genes included in it is a rate-limiting [26] CO_2_ fixation, which occurs in form of D-ribulose-1,5-bisphosphate carboxylation. This step involves ribulose-bisphosphate carboxylase enzyme. It consists of large and small subunits, each of which is present in eight copies in a functioning polypeptide. The large subunit contains active site of the enzyme and is chloroplast-encoded. In plants, a family of nuclear genes *RBCS* encodes different forms of small chain subunit that may be involved in regulation of enzyme catalytic activity [27]. It was also shown that translation level of large subunit is suppressed in absence of small subunit proteins [28].

In the current study, expression levels of gene MLOC_61558 that encodes rubisco large chain and is localized on morex_contig_456277 fragment of current genome assembly, as well as genes MLOC_44795, MLOC_21811 and MLOC_64679 that encode three rubisco small chain subunits, are shown to be lower in i:Bw*Alm* in comparison with Bowman. Differential expression was confirmed by EdgeR tool for genes MLOC_61558 and MLOC_21811 and by both EdgeR and cuffdiff tools for genes MLOC_64679 and MLOC_44795.

The following step in the pathway includes gene MLOC_76055, which encodes phosphoglycerate kinase (PGK), an enzyme that catalyzes phosphorilation of 3-phospho-D-glycerate. It exists in all living organisms and has a highly conserved structure. The enzyme functions as a monomer. In plants, PGK is reported to have two isozymes, one cytosolic and one chloroplast, which are encoded by two different nuclear genes and are expressed independently from each other [29].

The next step of Calvin-Benson-Bassham cycle is reduction of the carboxyl of 1,3-bisphosphoglycerate to an aldehyde. It involves enzyme glyceraldehyde-3-phosphate dehydrogenase (GAPDH). In plants, GAPDH exist in cytosole as a heterotetramer and in chloroplasts in a different form [30]. Subunits of this enzyme are encoded by nuclear genes [31]. PlantCyc database indicates that two genes are included into this step, one of which is MLOC_44511 gene that shows differential expression between studied lines of barley. Protein product of this gene is referred to as ‘predicted protein/uncharacterized protein’ in barley genome assembly version 1v28.

Differentially expressed genes MLOC_62317 and MLOC_19670 are included in regeneration of ribulose. This stage includes series of reactions and a variety of enzymes, among which is fructose-1,6-bisphosphatase enzyme, which is also involved in gluconeogenesis and sucrose biosynthesis.

Finally, gene MLOC_58999 that encodes phosphoribulokinase protein is involved in phosphorilation of D-ribulose-5-phosphate. Resulting product of the stage is D-ribulose-1,5-bisphosphate that is later used as a substrate by rubisco. However, differential expression for gene MLOC_58999 was not confirmed by either cuffdiff or EdgeR tools.

**Fig. 12:**
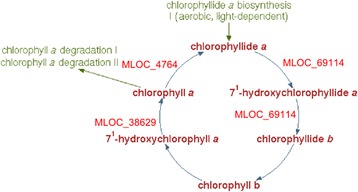
Involvement of differentially expressed genes in chlorophyll cycle pathway. The diagram of pathway was taken from BarleyCyc online database. Genes with differential expression are shown in red on the respective stages of their protein products involvement

**Fig. 13:**
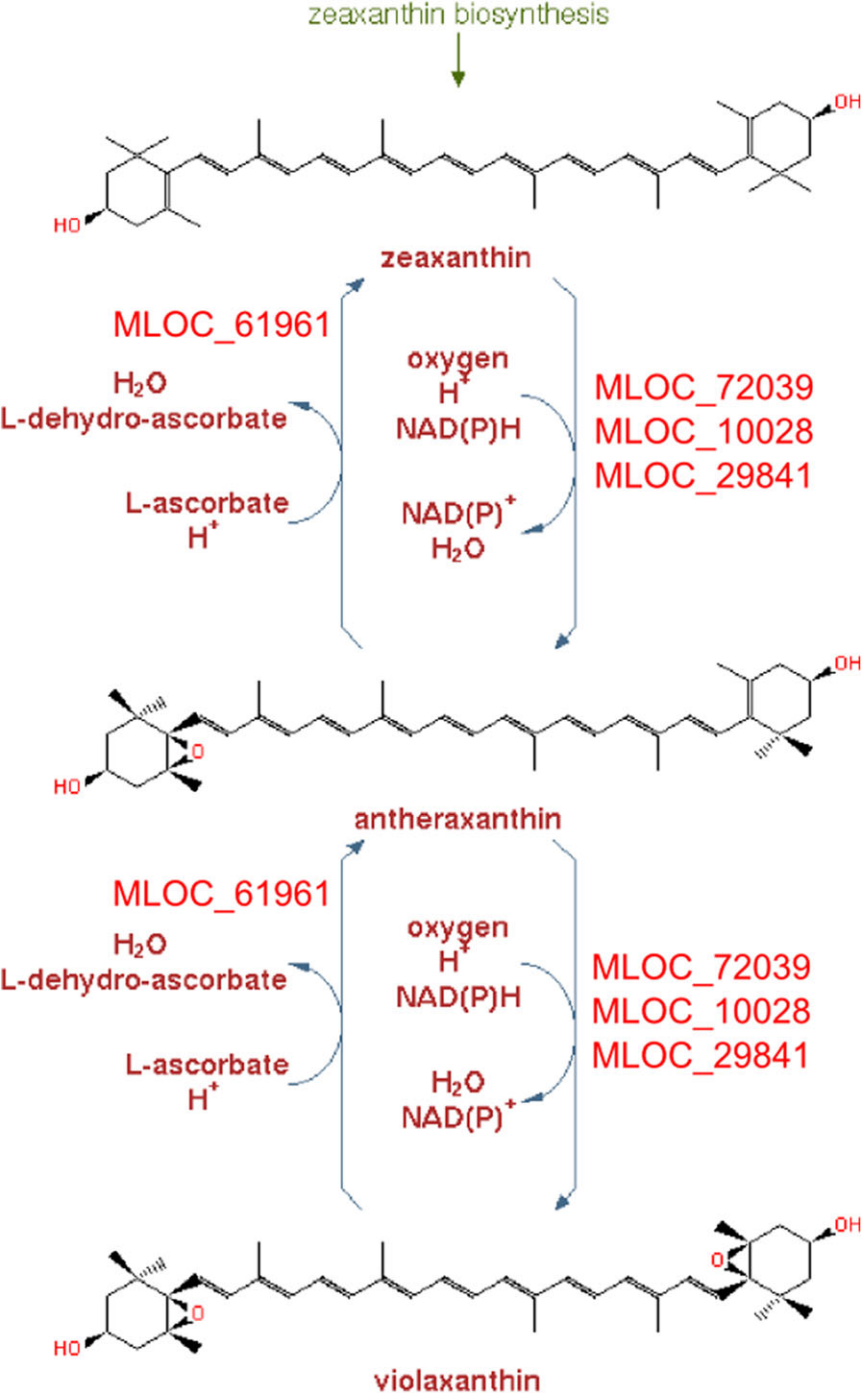
Involvement of differentially expressed genes in Xanthin cycle pathway. The diagram of pathway was taken from BarleyCyc online database. Genes with differential expression are shown in red on the respective stages of their protein products involvement

Furthermore, analysis of several plant pigment biosynthesis pathways demonstrated that they contain a number of differentially expressed genes. Pathways ‘Superpathway of anthocyanin biosynthesis (from delphinidin 3-O-glucoside)’ and ‘Superpathway of anthocyanin biosynthesis (from pelargonidin 3-O-glucoside)’ each have three barley genes annotated in PlantCyc database for *H. vulgare*. None of the genes demonstrated differential expression at the specified significance level. Pathway ‘Chlorophyll cycle’ contain five genes, three of them have significant changes in expression levels (Fig. 12). Pathway ‘zeaxanthin, antheraxanthin and violaxanthin interconversion’ has eight annotated genes; three of them have significant changes in expression level and one has differential isoform expression (Fig. 13). All differentially expressed genes included in these two pathways have a lower level of expression in i:Bw*Alm* line. Table 7 shows the lists of annotated barley genes included in the abovementioned pathways.

**Table 7:** Genes included in Chlorophyll cycle and Xanthin cycle pathways. Expression level changes and corrected *p*-values shown for genes with significant differential expression

Pathway	Gene name	Protein product	Log2(FC)	Corrected *p*-value
Xanthin cycle	MLOC_61961	non-photochemical quenching	−2.20	0.02
	MLOC_43271	violaxanthing de-epoxidase related		
	MLOC_72039	Unchar	−2.69	0.02
	MLOC_10028	chloroplastic lipocalin	−1.86	0.004
	MLOC_29841	Unchar	−2.66	0.006
	MLOC_74065	Unchar		
	MLOC_11534	Unchar		
	MLOC_64943.1	zeaxanthin epoxidase		
Chlorophyll cycle	MLOC_4764.2	chlorophyll synthase	−1.15	0.01
	MLOC_69114.2	71-hydroxychlorophyllide a monooxygenase	−4.48	3.74e-09
	MLOC_7670.1	chlorophyll(ide) b reductase		
	MLOC_71939.1	chlorophyll(ide) b reductase		
	MLOC_38629.3	MLOC_38629.3	−2.78	0.002

### De novo transcriptome assembly

*De novo* assembly of transcriptome was performed to verify transcriptome data for presence of novel transcripts. We merged data from different samples of the same genotype before assembly analysis. Statistics of resulted sequence assemblies provided in Table 8. Alignment of resulted transcripts to *H. vulgare* reference genome by megablast tool of BLAST package. A total of 565 and 605 transcripts in assemblies of iBw*Alm* and Bowman, respectively, showed no significant homology to any sequences of reference genome.

**Table 8:** Bowmanand i:Bw*Alm* libraries assembly statistics

Assembly	Transcripts, total	N50	Megabase assembled
All libraries	39620	949	28,9
i:Bw*Alm* libraries	86816	1112	66
Bowman libraries	107170	1150	86

Transcripts with no significant homology to barley genome were examined in details. They were mapped to *Escherichia coli* genome in order to remove possible contaminants. 29 transcripts from i:Bw*Alm* assembly and 28 transcripts from Bowman assembly showed homology with *E. coli* genome sequence. These transcripts were removed from further analysis. After aligning assembled transcripts without *H. vulgare* similarity to CDS sequences of *Oryza sativa* and *Brachypodium distachion* several homologous sequences were found. Alignment to *Arabidopsis thaliana* CDS sequences revealed no homology in any of the transcripts.

Thus, 564 transcripts from i:Bw*Alm* assembly and 604 transcripts from Bowman assembly showed no significant homology to *H. vulgare* genome (see additional files 7 and 8). Among those, 320 transcripts are common to both assemblies (Table 9).

**Table 9:** Mapping statistics of assembled transcriptomes

Assembly	Not mapped to *Hordeum* genome	Mapped to *Oryza* CDS	Mapped to *Brachypodium* CDS
I:Bw*Alm*	564	12	27
Bowman	604	23	35

## Discussion

### Differential expression analysis

1221 genome fragments were distinguished as having differential expression, while 1786 genome fragments have changes in expression level greater than four-folds, based on their RPKM values. In studies of plants comparative transcriptomics numbers of differentially expressed genes are usually presented in thousands: 3096 [32], 4811 [33], 5944 [34]. Furthermore, it is stated that no less than 20 % of plant genes express differentially in darkness and light [35, 36]. Thus, we suppose that a number of genes that we identified as differentially expressed should not be considered exaggerated. At the same time, changes in the transcriptional level do not necessarily correlate with changes in levels of translation [37], and not all the observed changes in transcription level of the genes may be functionally associated with phenotypic changes between two barley lines.

Five genes encoding different pentatricopeptide repeat-containing proteins (PPR) show changes in expression level, with four of them having lower level of expression in line i:Bw*Alm* and one having a higher level of expression in that line. PPR proteins are RNA-binding proteins that are especially abundant in land plants [38]. Most PPR proteins in plant cells are targeted either to mitochondria or chloroplast, where they bind RNA and affect gene expression [39]. More than 400 PPR genes are identified in *A. thaliana* genome [40], however, only 10 such genes are annotated in barley genome, which suggests that actual number of PPR genes that express differentially between Bowman and i:Bw*Alm* lines is much larger.

Gene ontology analysis revealed that genes with lower level of expression in line i:Bw*Alm* are mostly functionally associated with porphyrin pigment biosynthesis. Since chlorophylls a and b are chemical derivatives of porphyrin, it islikelythat these genes are indirectly associated with chlorophyll synthesis. Genes with higher level of expression in line i:Bw*Alm* are functionally associated with cytosol vesicles. Despite TIC and TOC membrane complexes being a primary path of protein transport into plastids [41],vesicular transport is known to play role in this process [42].

Pathway analysis showed that differentially expressed genes participate in several metabolic pathways, including photosynthesis light reaction and Calvin-Benson-Bassham cycle that occurs in chloroplasts. Interconversion of several xanthin derivatives is another biological process that takes place on chloroplast membranes [43]. Different forms of xanthins are included in light-harvesting complexes of chloroplast and participate in non-photosynthetic quenching process that protects the cell from oxydative stress [44]. Reduction in expression level of genes included in this pathway is in consistent with the general expression level decrease of genes functionally connected with chloroplasts. Likewise, observed lower expression levels of genes included in ‘Chlorophyll cycle’ pathway is in agreement with lowered amounts of chlorophyll in studied organs.

Variegated mutants that have patches of albino cells in leaves and other organs have been studied in regard of plastid composition of their cells that lack chlorophyll. It was shown that in many variegated mutants chloroplasts lack thylakoids but accumulate vesicles [45, 46].

### Plastid gene expression patterns in Bowman and i:Bw*Alm* genotypes

**Fig. 14:**
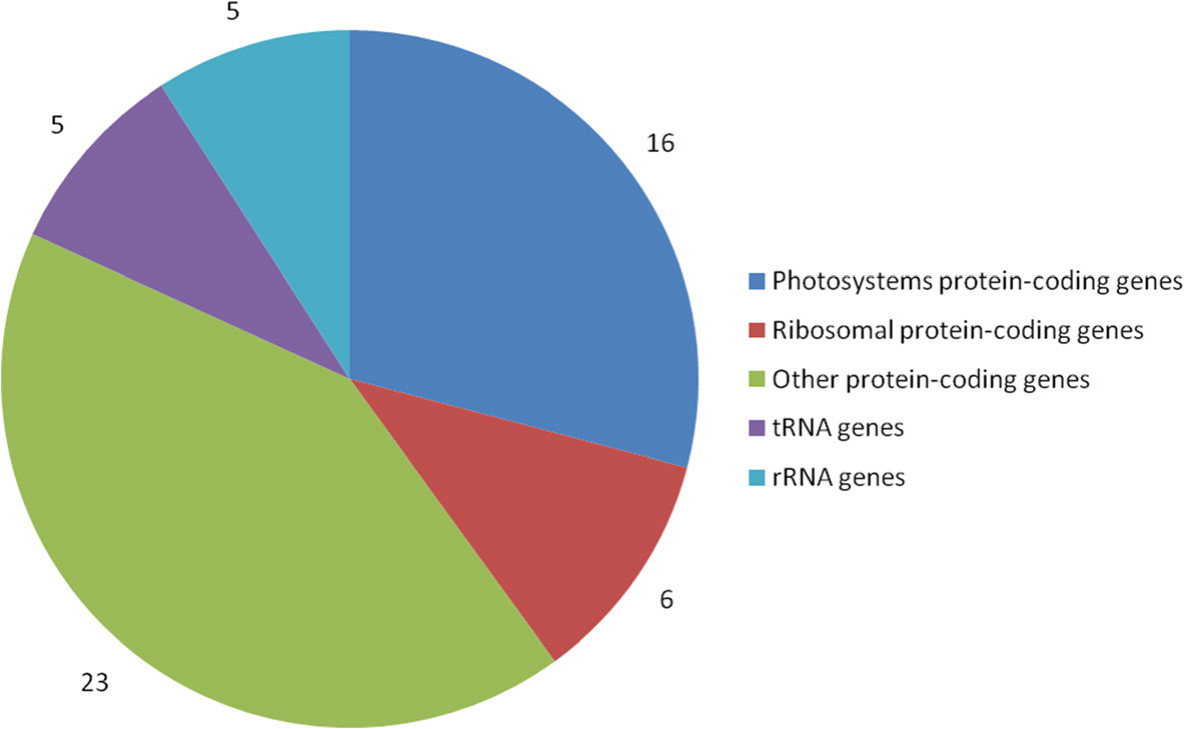
Distribution of functions of genes in differentially expressed plastid operons

Changes in transcript abundance of most plastid genome fragments are observed between two barley lines (suppl. file. 2). We did not observeany plastid genome fragment which abundance in line i:Bw*Alm* is increased compared to Bowman. The only genes that did not change their abundance in i:Bw*Alm*/Bowman are those having zero coverage in both barley lines. Changes in coverage observed for the majority of transcribed plastid genome fragments (20 of 47 transcribed genome fragments) is four-folds to sixteen-folds (2 < log_2_FC < 4). Majority (45 of 55) of genes in operons with changes in expression levels are protein-coding genes, mostly encoding ribosomal proteins (6 genes) and photosystems I and II proteins (16 genes) (Fig. 14). According to the results of microscopic analysis, many cells in studied organs lack chlorophyll. However, observed expression of several plastid genome fragments suggests that these cells still contain plastids, whether these are abnormal chloroplasts that do not contain chlorophyll or some other types of colorless plastids, presumably proplastids or etioplasts. At the same time, these plastids are either less abundant and/or less transcriptionally active than chloroplasts in cells of Bowman plants. This suggestion can explain overall decrease of plastid genome fragments coverage in line i:Bw*Alm*.

While most plastid genome fragments have a decrease in transcript abundance ranging from four-fold to sixteen-fold that can be accounted for by reduction in plastid number and/or general transcriptional activity, several genome fragments have a more abrupt decrease in transcription. Three genome fragments have decrease in transcript abundance of approximately 32 times in i:Bw*Alm* compared to Bowman. One of these fragments contains genes *psbC* and *psbD* encoding subunits of photosystem II, the other fragment contains a tRNA gene, and the third contains gene *psaC* encoding a photosystem I subunit and genes *ndhE* and *ndhD* that encode NADH-plastoquinoneoxydoreductase subunits. Gene *rps14* that encodes one of ribosome proteins has an 87-fold transcript reduction. Genes that encode 4.5S, 16S and 23S ribosomal RNAs have decrease in transcript abundance from 155-fold to 368-fold.

Finally, 15 genome fragments that are expressed in Bowman have zero coverage in i:Bw*Alm*. This list contains several genes encoding photosystems I and II subunits, NADH-plastoquinoneoxydoreductase subunits, tRNA genes, 5S rRNA gene and a gene encoding ATP synthase CF0 subunit IV.

It was shown that most plastid genes in *H. vulgare* belong to type II, i.e., have promoters for both PEP and NEP, with only *rpoB* gene being transcribed solely by NEP [47]. With this in mind, it looks surprising that we did not observe expression of *rpoB* and *rpoC2*genes in both lines. 23 genes encoding plastid tRNAs are not expressed in eitherlines as well. At the same time, plastidal rRNA-encoding genes have high levels of transcript abundance in both lines, except for 5S ribosomal RNA gene that shows no expression in i:Bw*Alm* line while being transcriptionally active in Bowman line.

Etioplast is a plastid that lacks chlorophyll and is colorless. Etioplasts form in leaves deprived of light. A comparison of chloroplast and etioplast proteomes revealed that several proteins present in etioplasts do not occur in chloroplasts [48]. Namely, protochlorophyllide reductase is believed to be an indispensable component of etioplast proteome. Gene *porA* encoding the enzyme is transcriptionally active both in i:Bw*Alm* and Bowman. However, its expression is mainly controlled on translation stage [49]. NAD(P)H dehydrogenase subunits I and J are present in etioplasts as well [48]. At the same time, in i:Bw*Alm* no transcripts of genes *ndhI* and *ndhJ* that encode two respective subunits were found, whereas a low level of transcription was observed for both genes in Bowman.

It should be noted, however, that changing the level of transcription for some genes maybe not the primary cause of albinism [50]. Albinism in plants may be due to various reasons, also including spontaneous chromosomal and ptDNA rearrangements, predominantly single or multiple deletions of DNA segments. Most deletions occur in the large single copy region, which is the least stable region in the entire plastome genome. This region primarily contains genes responsible for photosynthesis, genes encoding the proteins of photosystem I and II. Thus, further investigation is needed to clarify possible molecular mechanisms of in i:Bw*Alm* mutants in details.

### *De novo* transcriptome assembly

Three *de novo* transcriptome assemblies of three pooling combinations of six libraries were performed – (1) all six libraries together, (2) three Bowman libraries together and (3) three i:Bw*Alm* libraries together. As a result, assembly of all six pooled libraries was deemed ineffective, since total length of transcripts and N50 values in that assembly were inferior in comparison to other two assemblies. Therefore, assembly of all six pooled libraries was removed from further analysis.

More than 99 % of assembled transcripts were successfully mapped to current *H. vulgare* genome assembly. Furthermore, N50 for both assemblies is slightly higher than reported in other works for *H. vulgare* transcriptome assembly [51, 52]. This suggests that quality of assembly is satisfying. Relatively small number of transcripts that show no homology to barley genome and low percentage of short reads that did not map to genome during alignment point out that current version of *H. vulgare* genome, despite still being in development, is profound enough.

For sequences from *de novo* assembly that show no homology to genomic sequences of other plant species and have no replicates in the other assembly it can be assumed that they might be assembly errors. On the other hand, it is reported [53, 54] that other plant transcriptomes have a fraction of unique sequences that do not align to other plant genomes. A more thorough investigation of assembled transcripts is required to distinguish assembly errors from unique transcripts. The fact that no novel transcripts aligned to A*. thaliana* CDS suggests that novel transcripts contain sequences specific at least to *Poaceae*, and possibly even narrower group.

## Conclusions

Although peculiarities of genetic mechanisms controlling formation of albino lemma and pericarp phenotype remain unclear and require further investigation, several aspects of this process are clarified. Despite lack of chlorophyll and properly formed and functioning chloroplasts, albino cells retain transcriptionally-active plastids. While genes with lower level of expression in i:Bw*Alm* line are mostly associated with chlorophyll synthesis and photosynthesis, genes with higher level of expression in i:Bw*Alm* line are functionally associated with vesicle formation. Vesicles play essential role in transporting proteins to chloroplasts, and at the same time vesicles are accumulated in different types of non-photosynthesizing plastids, namely etioplasts and proplastids. Furthermore, it is known that in variegated leaves chlorophyll-lacking cells contain chloroplasts that accumulate vesicles as well.

Thus, possible variants of plastids inhibiting albino cells are proplastids, etioplasts or damaged chloroplasts that do not contain chlorophyll. Gene expression levels exhibit little correlation with protein composition of etioplasts. Further investigation is required in order to specify plastid numbers and structure in albino cells.

Despite being state-of-the-art method, RNA-seq and transcriptome analysis should not be implemented without aid from other techniques of investigation, such as microscopy and proteomic and metabolomic analysis. To uncover the mechanisms of partial albino phenotype formation in this particular case further examination is required, namely microscopic analysis in order to clarify plastid number, structure and location in albino cells. Since expression of most plastid genes is regulated on post-transcriptional stages, proteomic analysis can give insight into actual protein content of the albino cells and shed light into coordination between plastid genes transcription and actual levels of their protein products.

Finally, it is suspected that, since transition from proplastid to chloroplast occurs during organogenesis, transcriptomic analysis of a formed organ is unlikely to reveal genetic background of phenotype formation. To detect genetic mechanisms behind albino lemma and pericarp phenotype formation, earlier stages of organ development, at which chloroplast genesis in plants of Bowman line occurs, should be examined and compared.

## Methods

### Plant materials, DNA and RNA extraction, genotyping

*H. vulgare* cultivar Bowman and near-isogenic line i:Bw*Alm* (NGB20419) developed at Bowman background but carrying the *Alm* gene were grown in a hydroponic greenhouse at the shared center “Laboratory of Artificial Cultivation of Plants” of Institute of Cytology and Genetics SB RAS under a 14 h photoperiod. Plants were genotyped and used for RNA-seq analysis. DNA was extracted from leaf material using a procedure described in [55]. Primers amplifying the barley 3H microsatellite loci *Xgbms0022*, *Xgbms0046*, *Xgbms0048*, *Xgbms0050*, *Xgbms0085*, *Xgbms0102*, *Xgbms0149*, *Xgbms0212*, *Xebmac541, Xbmag225*, *Xbmag877* [56, 57] were used in PCRs conducted according to [58]. The resulting amplicons were visualized after separation through a 5 % agarose (ACTGene, Inc., Piscataway, NJ, USA) gel. The scheme of donor’s fragment in chromosome 3H of NGB20419 NIL is presented Additional file 9.

### Phenotypic characterization

*H. vulgare* cultivar Bowman and near-isogenic line (NGB20419) were compared visually, using transmitted light and chlorophyll fluorescence microscopy. Confocal images of chlorophyll fluorescence pattern in different plant organs were acquired with a laser scanning system LSM 780 NLO (ZEISS, Germany). Samples of freshly isolated plant fragments were placed on a glass slide and covered coverslip. Fluorescence emission spectra were acquired using the 405 nm ray line of a diode laser for excitation and the emitted fluorescence was detected from 550 to 700 nm. To represent the pattern of chlorophyll fluorescence from volumetric organs were performed maximum-intensity-projection processing using ZEN software (ZEISS, Germany).

### DNA and RNA extraction

Total RNA was extracted from i:Bw*Alm* developing spikelets with albino lemma and pericarp and from those of Bowman control using a Plant RNA MiniPrep^TM^ kit (Zymo Research Corporation, Irvine, CA, USA). RNA extracted from several plants was pooled together to exclude possible errors, introduced by deviations of biological material. Three biological replicates were prepared for each genotype.

### RNA preparation and sequencing

RNA was pooled into six libraries (three replicates for each line). Pooled libraries of short read were examined for quality of reads. Libraries were incubated with poly-T-tailed beads for poly-A enrichment. ERCC spike-in mix was added to each of libraries to create a form of built-in control. RNA in libraries was fragmented by incubation with nuclease enzymes. Sequencing was carried out with IonTorrent platform.

### qPCR experimental validation

For qPCR RNA was treated with DNAse (QIAGEN RNase-Free DNase Set). A 0.7 μg aliquot of RNA was used to prepare single-stranded cDNA by reverse transcription, based on a RevertAidTM kit (Thermo Fisher Scientific Inc., Waltham, MA, USA) and a (dT)15 primer. The subsequent qRT-PCR was based on a SYNTOL SYBR Green I kit (Syntol, Moscow, Russia). Type III LHCII CAB precursor protein gene and XLOC-012413 transcript abundance was assessed using the respective primer pairs (5’ CGACCAACGGCAGAATCAC 3’/5’ AGACGGGCTCCTTGAACTC 3’ and 5’ CATACTTGCTGCGTCCT 3’/5’ GAGTGGTCGTGTTCTGA 3’). The primers were designed using IDT PrimerQuest software (http://eu.idtdna.com/PrimerQuest/Home/). The reference sequence used was Ubc (ubiquitin), assayed using primers suggested in [59]. Three technical replicates of each reaction were run.

### Read preprocessing and mapping

We used FastQC (http://www.bioinformatics.bbsrc.ac.uk/projects/fastqc) to estimate sequencing quality, cutadapt [60] to remove adapter sequences and prinseq [61] to filter sequences by quality (minimal mean Phred quality 20) and length (minimal fragment length 50). Resulting filtered libraries were mapped to *H. vulgare* reference genome assembly 082214v1.28 from Ensembl Plants database.

We used TopHat2 [62] and STAR [63] tools for read mapping. Preliminary analysis was performed for these two mappers to obtain optimal results.

We run TopHat2 ten times increasing values of the ‘allowed mismatches’, ‘read edit distance’ and ‘read gap length’ parameters by 2 from 2to 20. Resulting alignments of TopHat2 were processed using Cufflinks [64] pipeline to obtain mRNA fragment counts and annotation. The following quality measures we estimated to choose the alignment parameters delivering maximal genome coverage and minimal mapping errors:

1. number of genome segments covered by reads continuously, N_cov_;
2. number of genome segments covered by single read only in all six libraries, N_orphans_;
3. number of annotated genome regions without library fragments aligned to them, N_uncov_;
4. proportion of library fragments aligned to the reference genome, F_mapped_.

STAR was used to align the libraries four times with the parameter ‘outFiletMismatchMax’ set at 4, 8, 12 and 18. A proportion of aligned library fragments was examined in order to investigate the influence of allowed number of mismatches. Increase in number of allowed mismatches did not result in significant increase in number of mapped reads. STAR alignments with 18 allowed mismatches were taken into further analysis.

To estimate the consistency of mRNA expression data from different libraries we normalized read counts obtained by Tophat method using cuffnorm. Mapping files of STAR were processed with ‘coverageBed’ utility of Bedtools [65] program to produce table of counts for each mapped fragment. Gff file based on genome marking produced by Cufflinks pipeline was used as a reference genome annotation in order to assess table of counts from STAR mappings. These estimates of the mRNA fragments expression were used as input to GeneCluster3.0 [66] software to cluster six libraries by UPGMA method using average clustering method. Reconstructed tree describing similarity of mRNA expression estimates in all six libraries was visualized using TreeView program [67].

### Gene expression analysis

Identification of differentially expressed genes was performed on TopHat2 mapping results by two methods: Cuffldiff and EdgeR [68]. To run Cufflinks pipeline we used default parameters. Genes were distinguished as differentially expressing if change of expression level was greater than two-fold (|log_2_FC| > 1) and *p*-value corrected through Benjamini-Hochberg procedure was less than 0.05 (q < 0.05). For EdgeR, differential expression detection was performed using Likelihood-ratio test. Genes were distinguished as diffrerentially expressed if the change of expression level was greater than two-fold and false discovery rate was less than 0.05 (|log_2_FC| > 1 and FDR < 0.05). Lists of differentially expressed genes with higher and lower levels of expression in i:Bw*Alm* line were then analyzed separately. Resulting lists of differentially expressed genes obtained by cufflinks pipeline and EdgeR tool were compared to each other. Further analysis was performed on the list of genes with significant differential expression confirmed with either of tools, while keeping tracks for each gene in respect to which tool its differential expression was confirmed with.

Isoform differential expression was computed with Cufflinks pipeline. Cufflinks forms lists of potential isoforms for each transcribed genome fragment based on data on exon-intron structure annotated in the genome assembly. Genes were distinguished as having differential isoform expression if they have differential expression with the criteria listed above of two isoforms in two studied lines.

### Transcript functional annotation

Prior to the transcriptome sequence annotation, we assigned Barley affymetrix array sequences to the list of differentially expressed transcripts with higher and lower levels of expression in line i:Bw*Alm* separately by sequence similarity using Blast software. Gene ontology classification and enrichment analysis was performed by AgriGO online service [69].

A list of genes taken for pathway analysis consisted of genes annotated in the *H. vulgare* genome assembly and having changes of expression level greater than four-fold as derived from their FPKM values across all twelve mappings. The pathway analysis was performed using PlantCyc (http://www.plantcyc.org/) database. ‘Overlay experimental data’ option was selected for the Cellular Overview of *H. vulgare* (http://pmn.plantcyc.org/overviewsWeb/celOv.shtml). Experimental data was provided in form of text file with column of gene identifier and column with values of log_2_ of FPKM change for respective genes between the two barley lines.

In addition, BarleyCyc database was examined specifically for pathways of plant pigments biosynthesis. Following pathways were studied: ‘Superpathway of anthocyanin biosynthesis (from delphinidin 3-O-glucoside)’, ‘Superpathway of anthocyanin biosynthesis (from pelargonidin 3-O-glucoside)’, ‘Chlorophyll cycle’ and ‘zeaxanthin, antheraxanthin and violaxanthin interconversion’ that is a part of ‘superpathway of carotenoid biosynthesis’. ‘Customize or Overlay Omics Data on Pathway Diagram’ option was implemented, and list of differentially expressed genes was used to overlay on the list of genes included in the pathway.

### De novo transcriptome assembly and analysis

*De novo* assembly of transcriptome was conducted using Trinity tool [70]. All libraries for each biological case were pooled together, and resulting sets of fragments were assembled using default parameters. Each assembly was aligned to *H.vulgare* reference genome using Blastn tool of blast suite with [71]. Reliability threshold for homology detection was e < 10^−10^. Fragments that had no significant homology with reference genome were aligned to *Echerichia coli* genome (NCBI reference sequence NC_000913.3) in order to remove possible contaminants. After removing contaminants, remaining contigs were aligned to *Brachypodium distachion* CDS library version 1.0.28, *Oryza sativa* CDS library version 1.0.28and *Arabidopsis thaliana* CDS library of version TAIR10. *O. sativa* and *B. distachion* sequences were retrieved from Ensembl Plants database, *A. thaliana* sequences were obtained from TAIR database [72]

## Additional files

Additional file 1 Table of FPKM values for each transcribed genome fragment for each of six libraries, as derived from TopPat2 mappings; lists of differentially expressed genes obtained with EdgeR and Cufflinks tools. (XLSX 3393 kb)
Additional file 2 Plastid genome expressed fragments with respective annotated genes, location in plastid genome, FPKM levels for lines i:Bw*Alm* and Bowman and log_2_ for expression level fold changes. (XLSX 17 kb)
Additional file 3 Table of differentially expressed gene isoforms as derived from Cufflinks pipeline results. (XLSX 11200 kb)
Additional file 4 Ontology terms associated with biological process of protein products for genes with lower expression level in i:Bw*Alm* line. (PDF 89 kb)
Additional file 5 Ontology terms associated with cellular localization of protein products for genes with lower expression level in i:Bw*Alm* line. (PDF 185 kb)
Additional file 6 Ontology terms associated with cellular localization of protein products for genes with higher expression level in i:Bw*Alm* line. (PDF 52 kb)
Additional file 7 Sequences generated by Trinity from i:Bw*Alm* libraries that did not align to *H. vulgare* reference genome. (FASTA 325 kb)
Additional file 8 Sequences generated by Trinity from Bowman libraries that did not align to *H. vulgare* reference genome. (FASTA 337 kb)
Additional file 9 Chromosome 3H scheme of Bowman (left) and NGB20419/i:Bw*Alm* (right). *Alm1* donor segment on chromosome 3H remaining in the i:Bw*Alm* NIL, revealed by microsatellite genotyping, is in gray. (PDF 267 kb)

## Abbreviations

CRM: Chloroplast RNA splicing and ribosome maturation proteins
FPKM: Fragments Per Kilobase per Million of reads
GAPDH: Glyceraldehyde-3-phosphate dehydrogenase
GO: Gene ontology
JRL: Jacalin-Related Lectin
NADP-ME: Nicotinamide adenine dinucleotide phosphate malic enzyme
nsLTP: non-specific Lipid Transfer Protein
PGK: Phosphoglycerate kinase
PPR: Pentatricopepride repeat protein
TIC/TOC: Translocon on the inner/outer chloroplast membrane
TPR: Tetratricopeptide repeat protein
