# Supplementary figures and images for: Identification of nuclear genes controlling chlorophyll synthesis in barley by RNA-seq
Source: BMC Plant Biol. 2016 Nov 16;16(Suppl 3):119–38. doi: 10.1186/s12870-016-0926-x (PMC5123340; doi:10.1186/s12870-016-0926-x)

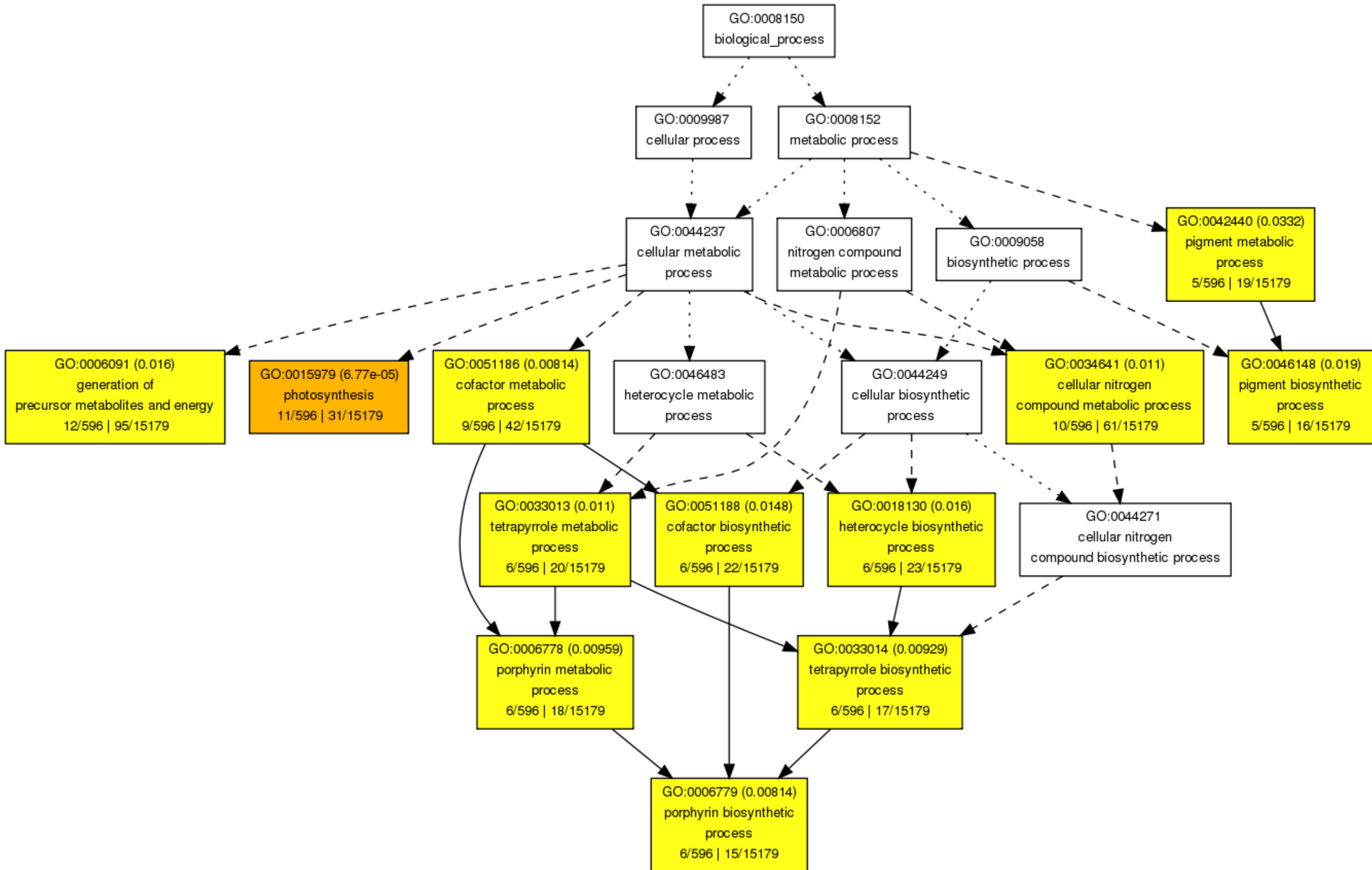

Supplement: Additional file 4 — Ontology terms associated with biological process of protein products for genes with lower expression level in i:BwAlm line. (PDF 89 kb) [file 12870_2016_926_MOESM4_ESM.pdf]

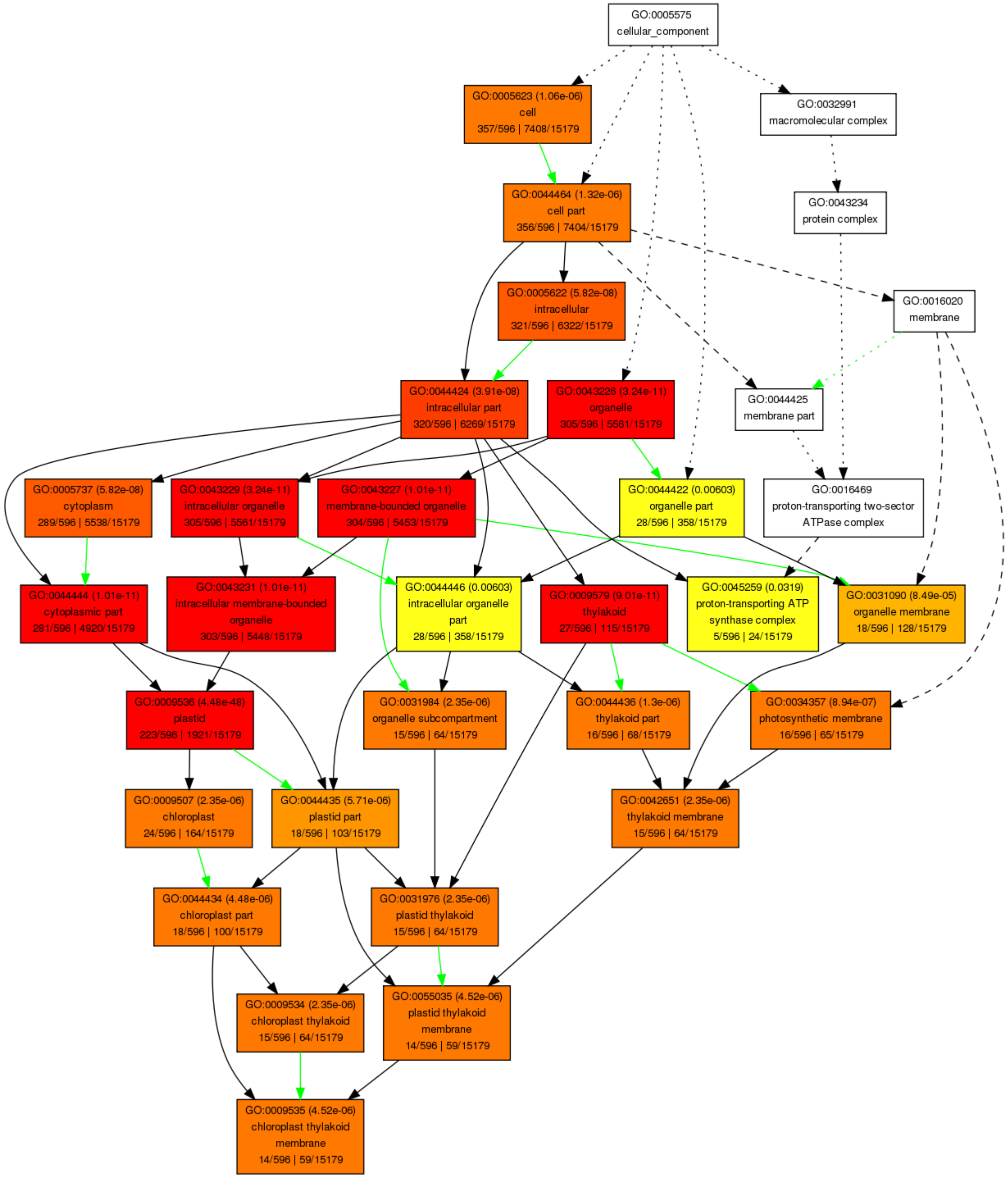

Supplement: Additional file 5 — Ontology terms associated with cellular localization of protein products for genes with lower expression level in i:BwAlm line. (PDF 185 kb) [file 12870_2016_926_MOESM5_ESM.pdf]

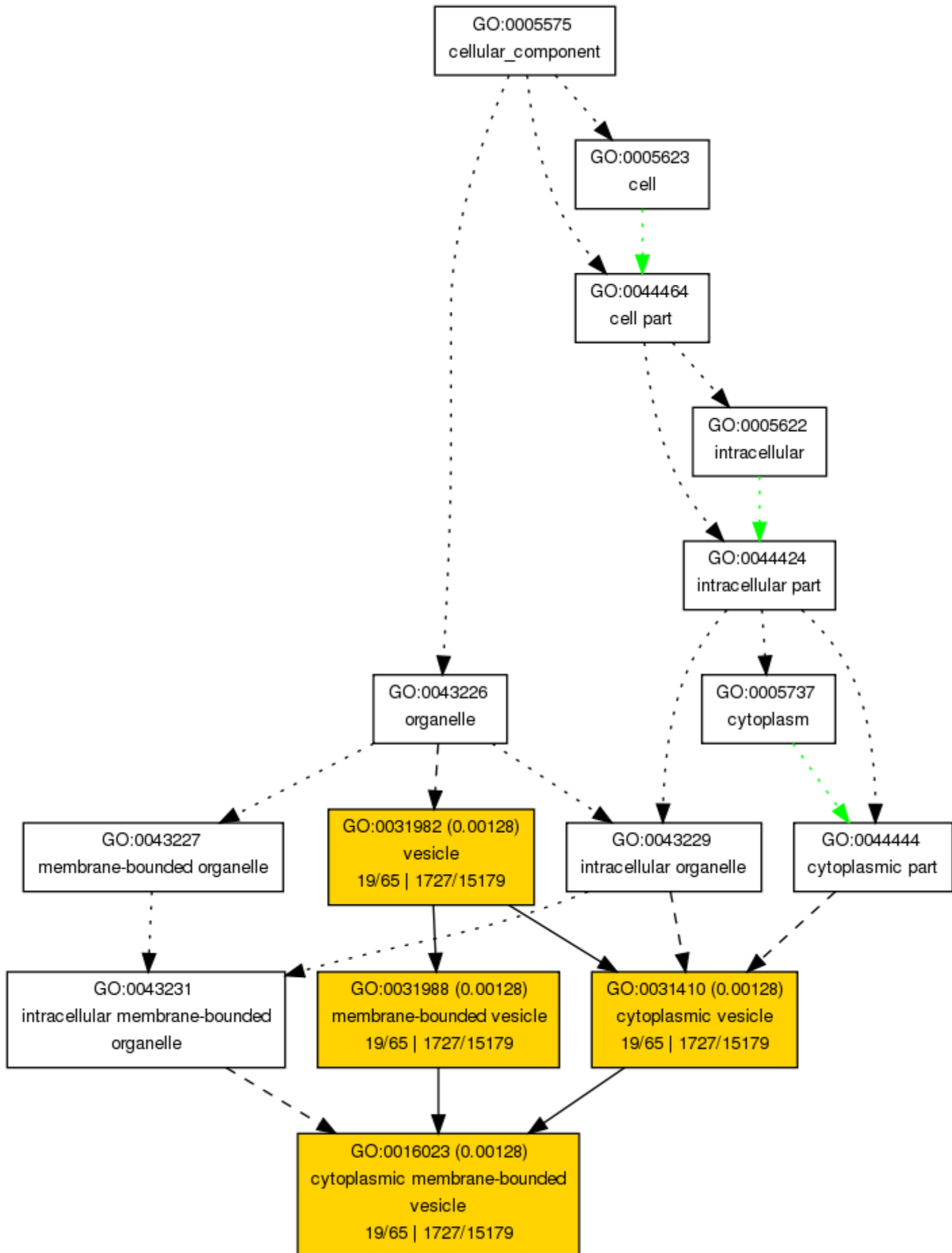

Supplement: Additional file 6 — Ontology terms associated with cellular localization of protein products for genes with higher expression level in i:BwAlm line. (PDF 52 kb) [file 12870_2016_926_MOESM6_ESM.pdf]
