# Supplementary material for: Identification of nuclear genes controlling chlorophyll synthesis in barley by RNA-seq
Source: BMC Plant Biol. 2016 Nov 16;16(Suppl 3):119–38. doi: 10.1186/s12870-016-0926-x (PMC5123340; doi:10.1186/s12870-016-0926-x)
Supplement: Additional file 9 — Chromosome 3H scheme of Bowman (left) and NGB20419/i:BwAlm (right). Alm1 donor segment on chromosome 3H remaining in the i:BwAlm NIL, revealed by microsatellite genotyping, is in gray. (PDF 267 kb) [file 12870_2016_926_MOESM9_ESM.pdf]

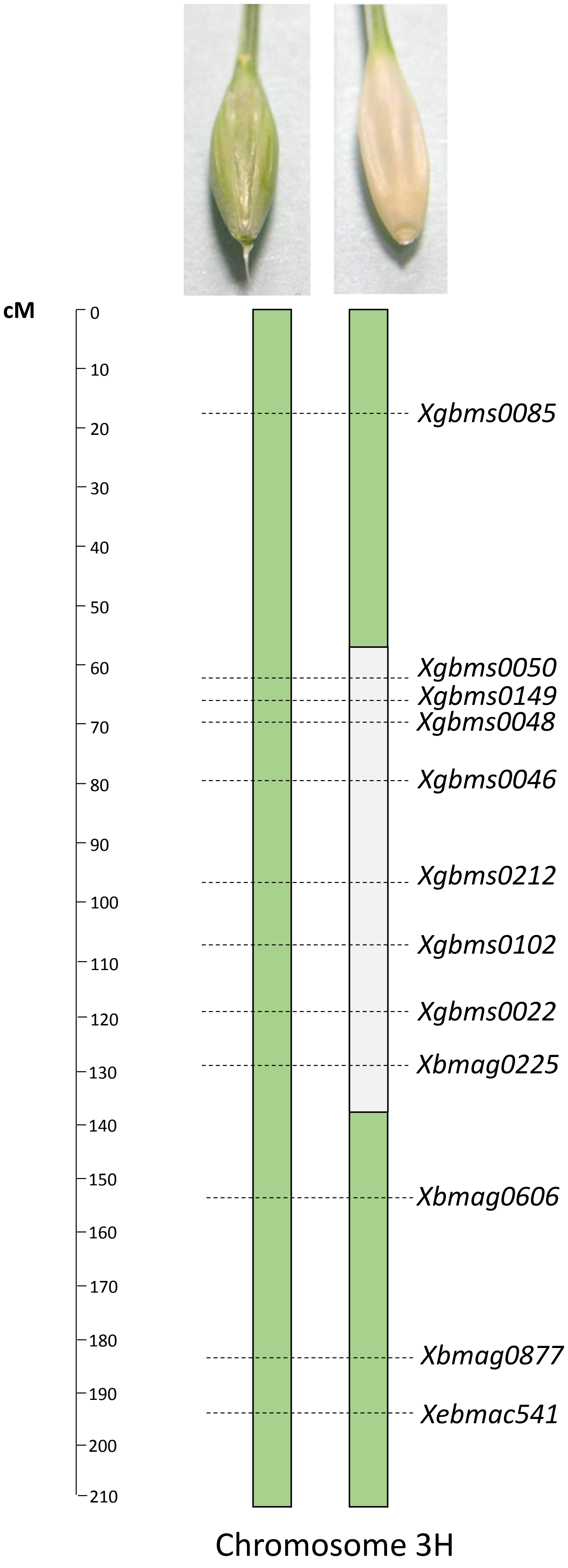

Chromosome 3H scheme of Bowman (left) and NGB20419 / i:Bw*Alm* (right). *Alm1* donor segment on chromosome 3H remaining in the i:Bw*Alm* NIL, revealed by microsatellite genotyping, is in gray.
